# The complete chloroplast genome sequence of Red Asparagus Lettuce (*Lactuca sativa* var. *asparagine* L. *red*) (Asteraceae), endemic to China

**DOI:** 10.1080/23802359.2020.1778553

**Published:** 2020-07-25

**Authors:** Ming-Xia Liu, Xing-Ling Tao, Hui-Xia Zhu, Dong Hou

**Affiliations:** Vegetable Research Institute, Gansu Academy of Agriculture Sciences, Lanzhou, China

**Keywords:** Red Asparagus Lettuce, *Lactuca sativa* var. *asparagine* L. *red*, chloroplast genome, phylogenetic analysis

## Abstract

Red Asparagus Lettuce (*Lactuca sativa* var. *asparagina* L. *red*) is an endemic vegetable species to China. The complete chloroplast (cp) genome of it is 152,744 bp in size and comprises a pair of inverted repeat regions of 25,033 bp each, a large single-copy (LSC) region of 84,103 bp and a small single-copy (SSC) region of 18,575 bp. A total of 131 genes were annotated in the cp genome, including 86 protein-coding genes, 8 ribosomal RNA genes, and 37 transfer RNA genes. The overall GC content of the Red Asparagus Lettuce cp genome was 37.55%. Phylogenetic analysis indicated that Red Asparagus Lettuce was more phylogenetically related to *L. sativa*.

Red Asparagus Lettuce (*Lactuca sativa* var. *Asparagina* L. *red*) is an endemic vegetable species to China, which is a variety of lettuce (*Lactuca sativa*), grown for its thick, succulent, edible stems, an annual plant of the daisy family, Asteraceae. It is a rich source of micronutrients (vitamin K, vitamin A, folate, iron), and is chock-full of anthocyanins, which prevent tumors from forming and suppress their growth, is becoming increasingly popular in China (Liu et al. 2016). However, due to the special floral structure, the central stigma pushes through a crown of stamens and automatically is pollinated. Therefore, the breeding of red asparagus lettuce is very difficult . Good knowledge of its genetics would contribute to the formulation of breeding and the study of genome diversity and species diversity. In this study, we reported the complete sequence of the chloroplast (cp) genome of Red Asparagus Lettuce for the first time and constructed the phylogeny of Asteraceae species based on the available complete cp genome sequences. The complete cp genome sequence of Red Asparagus Lettuce has been deposited in GenBank under the accession number ‘MT162684.’

The whole genomic DNA of Red Asparagus Lettuce was extracted from fresh leaves of an individual collected from Lanzhou (Gansu, China; 36°05′N, 103°43′E). A voucher specimen is held in the Vegetable Research Institute, Gansu Academy of Agriculture Sciences (Lanzhou, China), the specimen Accession number is V09FW1-1. The whole-genome sequencing was conducted by Nanjing Gene Pioneer Biotechnologies Inc. (Nanjing, China) on the Illumina Hiseq platform. The filtered sequences were assembled using the program SPAdes assembler 3.10.0 (Bankevich et al. 2012). Annotation was performed using the DOGMA (Wyman et al. 2004). Genome alignment was conducted with BLAT (Kent 2002). The cp genome was annotated using the software CpGAVAS (Liu et al. 2012), and the plastid genome map was generated by OGDRAW (Lohse et al. 2013).

The complete cp genome sequence of Red Asparagus Lettuce (Genbank accession number MT162684) contains 152,744 bp, with 84,103 bp in the large single-copy (LSC) region and 18,575 bp in the small single-copy (SSC) region, and 25,033 bp in the inverted repeat (IR) region. A total of 131 genes were annotated in the cp genome, including 86 protein-coding genes, 37 tRNA genes, and 8 rRNA genes. There are 7 protein-coding genes, 7 tRNA genes, and 4 rRNA genes that were duplicated in the IR region. 17 genes contained one intron and two genes, *clpP* and *ycf32* contained a couple of introns. The overall GC content of Red Asparagus Lettuce cp genome was 37.55%, while it was 43.03% of IRs, which was higher than LSC and SSC regions (35.73 and 31.05%, respectively).

To confirm the phylogenetic position of Red Asparagus Lettuce, whole cp genomes from 13 Asteraceae plants were aligned by MAFFT version 7 (Katoh and Standley 2013). A maximum-likelihood (ML) tree was reconstructed based on FastTree version 2.1.10 (Price et al. 2010). The ML phylogenetic tree shows that Red Asparagus Lettuce is closely related to *Lactuca sativa* (Figure 1). This annotated cp genome laid a good foundation for population genomic studies and genetic engineering investigations for this endemic vegetable species.

**Figure 1.**
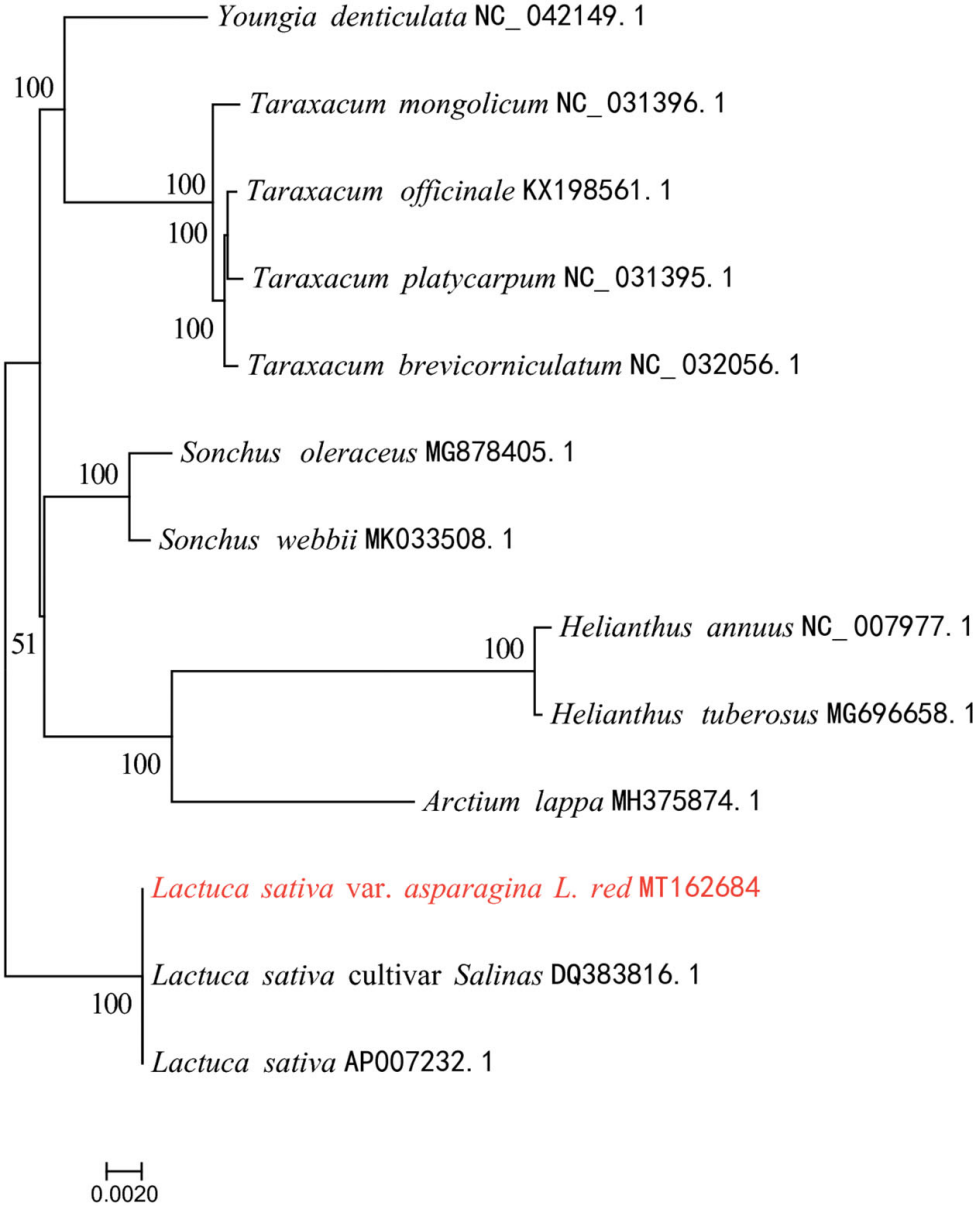
Maximum-likelihood phylogenetic tree inferred for Red Asparagus Lettuce from 13 cp genomes of Asteraceae plants. The bootstrap-ping values are listed for each node.

## Data Availability

Raw sequencing reads of Red Asparagus Lettuce (*Lactuca sativa* var. *Asparagina* L. *Red*) accessions reported in this study have been deposited into the National Center for Biotechnology Information GenBank (https://www.ncbi.nlm.nih.gov/Genbank/update.html) database under accession number of MT162684.
